# Characterization of infectious bacterial keratitis in Östergötland County, Sweden: a 10-year retrospective study

**DOI:** 10.1186/s12348-024-00432-y

**Published:** 2024-10-07

**Authors:** Jenny Roth, Baris Toprak, Sofia Somajo, Antonio Filipe Macedo, Neil Lagali

**Affiliations:** 1 Department of Medicine and Optometry, Linnaeus University, Kalmar, 39182 Sweden; 2 Department of Biomedical and Clinical Sciences, Faculty of Medicine, Linköping University, Linköping, 581 83 Sweden; 3 Department of Chemistry and Biomedical Sciences, Linnaeus University, Kalmar, 39182 Sweden; 4 Department and Centre of Physics-Optometry and Vision Science, University of Minho, Braga, Portugal

**Keywords:** Antibiotic susceptibility, Keratitis, Bacteria, Aetiology

## Abstract

**Background:**

The aim of this study was to characterize bacterial species, aetiology and antibiotic susceptibility connected to bacterial keratitis infections in Östergötland, Sweden.

**Methods:**

Retrospective cross-sectional study based on electronic health records for the period 2010–2019. Records of patients diagnosed with infectious keratitis were screened for microbiology confirmed infectious bacterial keratitis. Bacterial species and their susceptibility to antibiotics were determined from microbiology test results.

**Results:**

One-hundred and ninety patients with lab culture-confirmed infectious bacterial keratitis were included in the analysis. The most frequently found bacterial species were coagulase-negative staphylococci (39%), *Staphylococcus aureus* (17%) and *Cutibacterium acnes* (10%). *Pseudomonas* spp. was the most frequently found Gram-negative bacterial species (7%). Contact lens wear and severely ill/blind eye were the top two aetiologies associated with bacterial keratitis, 22% of the patients with bacterial keratitis were also diagnosed with glaucoma. Most isolates, 157 out of 173, were susceptible to fluoroquinolones, and 145 out of 155 isolates were susceptible to chloramphenicol.

**Conclusion:**

Our results revealed a positive rate of bacterial keratitis of 59% for the samples sent to the laboratory. There was a high susceptibility of the bacterial species to the recommended antibiotics. Our results indicate that it is likely that patients are receiving the correct treatment. Future studies are necessary to monitor changes in antibiotic susceptibility.

## Introduction

Infectious keratitis (IK) can be caused by different pathogens including bacteria, viruses, fungi, or parasites and is the leading cause of cornea-related blindness globally [1–3]. Bacterial keratitis (BK) is the predominant type of IK in cold climates of Europe and North America [1, 4]. This contrasts with countries with warmer climates, for example, in Southeast Asia, where fungal infections are the most frequent [1, 5]. The widespread use of contact lenses has been linked to an increased occurrence of contact lens-related keratitis especially in North America and Europe [6–8]. A study from Sweden revealed that contact lens wear was the most common risk factor for IK, with 45% of patients with IK reporting contact lens wear prior to the infection [9], it is unclear from the study results if other conditions like glaucoma were also prevalent in the sample.

Glaucoma, in most cases, requires life-long treatment with eye drops [10]. Up to 78% of patients with glaucoma also suffer from ocular surface disease (OSD), which can be caused by surface toxic components of glaucoma eye drops [11]. A compromised ocular surface increases the risk of infections in people diagnosed with glaucoma [12]. Also, it has been reported that bottles with eye drops for glaucoma treatment can have bacterial contamination rates between 10% and 60% [13], contamination has been found even in sealed bottles [14]. In the current study we paid special attention to cases with both glaucoma and keratitis.

In Sweden, the first line of treatment for BK consists of broad-spectrum topical antibiotics such as fluoroquinolones and chloramphenicol [15]. When the infection is severe, treatments should be guided by results from bacterial cultures and antimicrobial susceptibility profiling [16, 17]. The widespread use of fluoroquinolones in the treatment of systemic and ocular disease has been associated with increased levels of fluoroquinolone resistance in some regions [18–22]. It is, therefore, necessary to perform local epidemiological surveillance to use the correct antibiotics for the initial empirical treatment [23]. The current study has been designed to address the lack of updated surveillance data from Sweden with a comprehensive analysis of the susceptibility to antibiotics in bacterial species associated with infectious BK.

The aim of the current 10-year (2010–2019) retrospective study was twofold: (i) to identify bacterial species causing BK and aetiology associated with the infection; and (ii) to determine the susceptibility to antibiotics (aka antibiotic resistance) among the bacterial species causing BK in an university-hospital in Östergötland County in Sweden.

## Materials and methods

### Ethics

The study protocol was reviewed and approved by the Swedish Ethics Authority (Etikprövningsmyndigheten, protocol number 2019–05234) and was conducted in accordance with the principles of the Declaration of Helsinki.

### Study population

We used the electronic health records to identify all patients with any type of keratitis treated between 2010 and 2019 within Östergötland County. We exported from the records information from all cases with one or more of the following ICD10 diagnosis codes: H16: Keratitis, H16.1: Other superficial keratitis without conjunctivitis, H16.2: Keratoconjunctivitis, H16.3: Interstitial and deep keratitis, H16.8: Other keratitis, H:16.9: Keratitis, unspecified, H19.2: Keratitis and keratoconjunctivitis in other infectious and parasitic diseases classified elsewhere, H19.3: Keratitis and keratoconjunctivitis in other diseases classified elsewhere.

*First manual selection*: medical journals of the cases identified from the electronic records were reviewed by ophthalmologist and co-author Baris Toprak (BT). Cases with suspected BK were included in the study and cases with other types of keratitis were excluded. *Second manual selection*: to remain included in the study, BK cases had to have a microbiological investigation, and those records were also confirmed by BT. The microbiological investigation was performed according to the guidelines of the Swedish Ophthalmology Society [15], that is, for cases with corneal ulcers with a diameter of 1 mm or more or atypical presentation of corneal ulcer, such as engagement of deeper layers of cornea. Samples were collected with direct and indirect inoculation of the corneal specimen. Conjunctival samples were taken with the indirect inoculation method, details are given in appendix A. *Third manual selection*: only cases (patients) with a culture-confirmed BK were included in the final analysis.

### Culture records

Culture results retrieved from the clinical microbiology laboratory included information about bacterial species, sub-species identifications and results from antimicrobial susceptibility testing. Bacteria are reported as individual species, as sub-species or as group of bacteria– further details are given in appendix B.

Tested antibiotics were assigned into the antimicrobial classes: aminoglycosides (tobramycin, gentamicin), fluoroquinolones (ciprofloxacin, levofloxacin, norfloxacin), beta-lactams - cephalosporins of 1st (cefadroxil), 2nd (cefuroxime, loracarbef), 3rd generation (cefotaxime, ceftazidime) and carbapenems (imipenem, meropenem). Results for chloramphenicol and vancomycin are reported separately.

Antimicrobial susceptibility information was available according to the SIR categorization (S-susceptible; I-intermediate; R-resistant). There was no information regarding the minimum inhibitory concentration, zone diameters or use of screening agents. The interpretation to classification SIR had been made by the laboratory following local guidelines which rely on valid versions of the European Committee on Antimicrobial Susceptibility Testing breakpoint tables (including topical agents from 2014 and onwards).

### Statistical analysis

Statistical analysis was performed with SPSS Version 29.0 (IBM Inc., Armonk, NY). We compared counts with chi-square tests and risks with odds ratio (OR). A p-value of less than 0.05 was considered statistically significant.

## Results

**Fig. 1:**
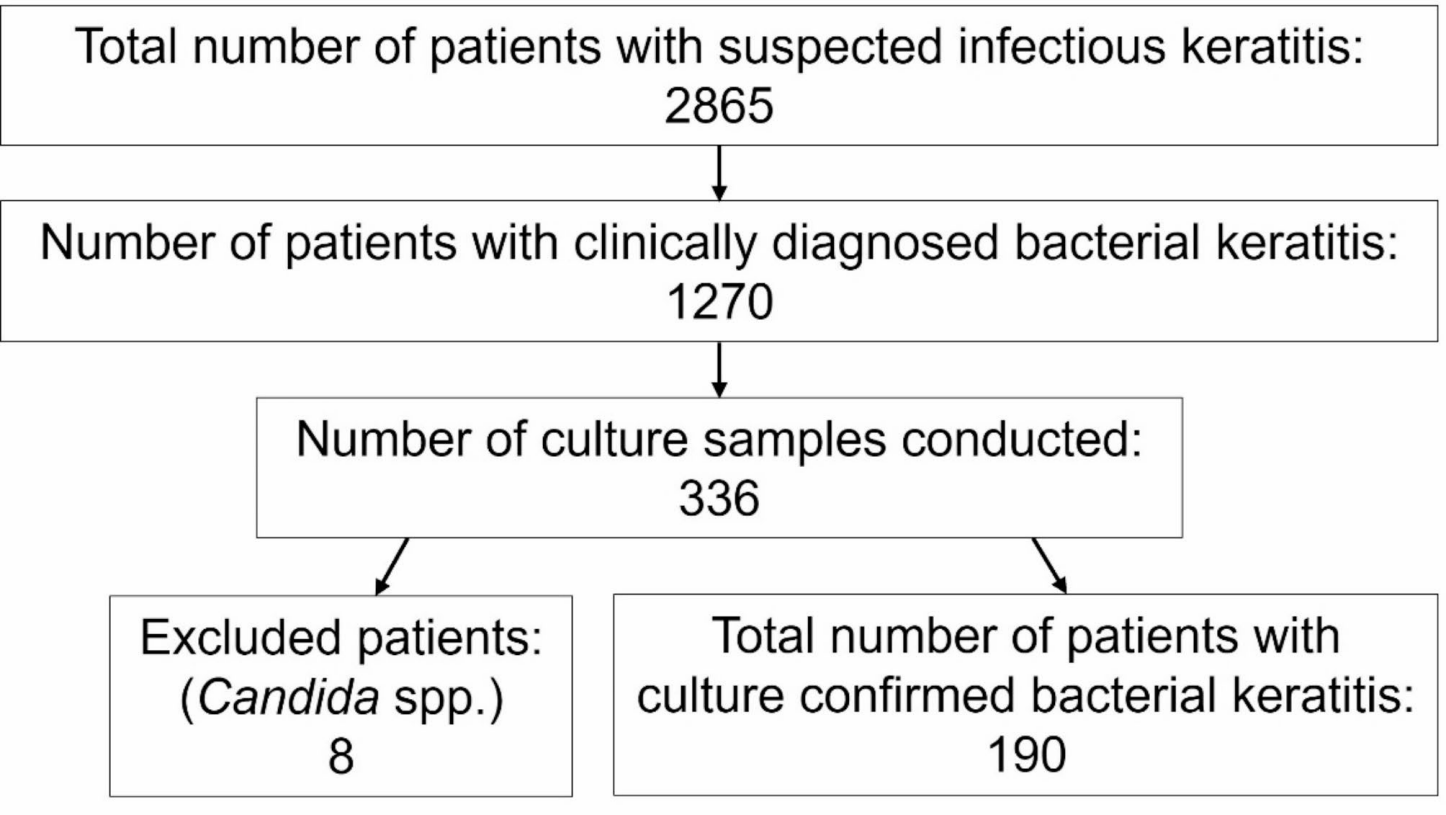
Flowchart summarizing the selection process of the cases investigated and reported in the current manuscript

The initial search retrieved 2865 records with suspected IK, the region has a population of approximately 1 million people. Figure 1 shows the process of selection of the data analysed in the current study. BK was clinically diagnosed in 1270 patients, samples from 336 patients were sent to the clinical microbiology laboratory for culture confirmation as per guidelines. The laboratory analysis revealed 190 patients with cultured confirmed BK and 8 patients with *Candida* spp. The *Candida* spp. patients were excluded from further analysis as indicated in Fig. 1, patients with combined fungal and bacterial infection were included. The culture positive rate was 59%. The laboratory tests yielded 224 bacterial findings from a total of 170 samples taken from the cornea and 54 taken from the conjunctiva. The counts of the bacterial species from cultures taken from cornea and conjunctiva were compared and we failed to find statistically significant differences in species profile between the two sampling sites (*chi-square test*, *p* = 0.087).

**Fig. 2:**
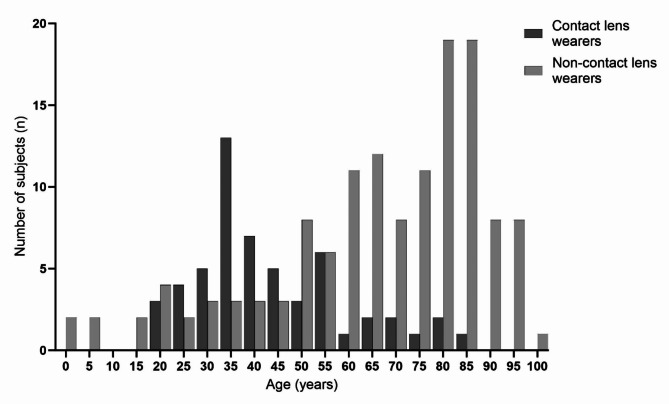
Age distribution of contact lens wearers and non-contact lens wearers, showing a peak number of subjects at clearly differing ages

For the 190 patients with positive BK the age ranged from under 1 to 99 years (median 62 years, IQR 40–79), 104 (55%) were females. Further analysis of demographics revealed a two-peaked age distribution in the sample shown in Fig. 2. Contact lens wearers with a median age of 39 years (IQR = 33–53) were significantly younger than the rest of the participants (median age = 71 years, IQR = 54–84, *P* < 0.0001). Out of 190, 156 (82%) patients were positive for growth of a single bacterial species, 25 (13%) patients were positive for two bacterial species and 7 (4%) patients were positive to three different bacterial species. Two patients were positive for one bacterial and one fungal species simultaneously (1%).

**Fig. 3:**
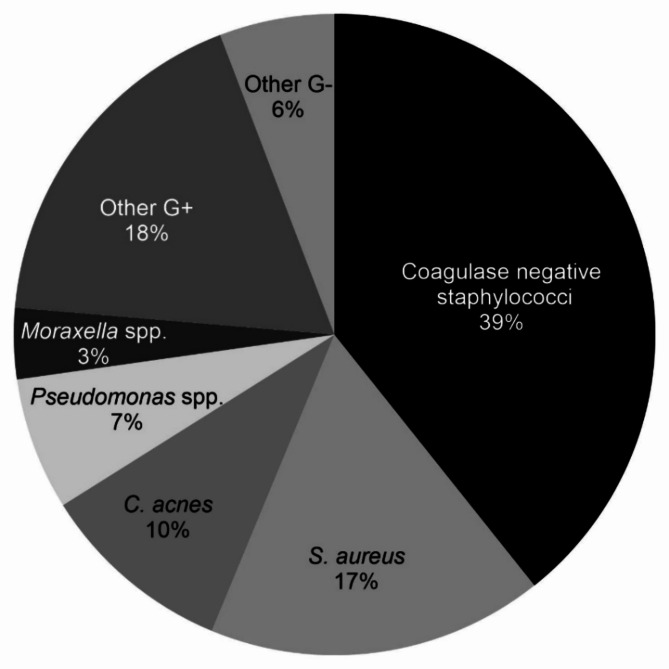
Distribution of laboratory reported organisms in the 190 patients with bacterial keratitis

The microbiology laboratory results for the combined bacterial and aetiology findings are summarized in Table 1; Fig. 3. Of a total of 224 bacterial findings, 188 (84%) were Gram-positive and 36 (16%) were Gram-negative. The most frequently reported bacteria were species from the group CoNS (39%), *S. aureus* (17%) and *C. acnes.* (10%). Among the Gram-negative bacteria, *Pseudomonas* spp. was the most frequently reported finding (7% of the total number of species and 42% of the Gram-negative species) followed by *Moraxella* spp. (3%).

**Fig. 4:**
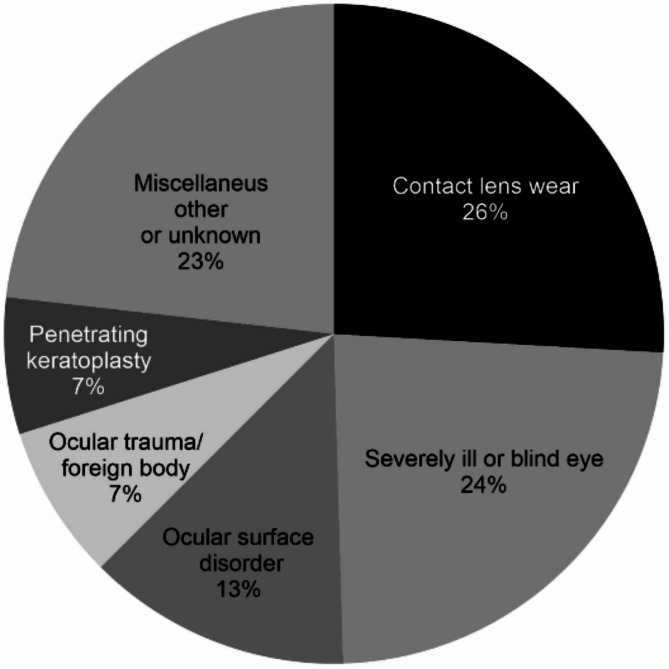
Distribution of clinical aetiology in the 190 patients with bacterial keratitis

Results given in Table 1; Fig. 4 show that the most common aetiology with 58 species (26% of the total) were identified among contact lens wearers and, therefore, can be considered as contact lens-related keratitis. CoNS was the most common Gram-positive pathogen in contact lens wearers corresponding to 27 (61%) of the 44 Gram-positive pathogens found and was significantly more common than other Gram-positive species (chi-square test, *p* < 0.001). *Pseudomona*s spp. was the most common Gram-negative pathogen, found in 10 (71%) out of the 14 Gram-negative pathogens, and was also found more frequently than other Gram-negative species (chi-square, *p* < 0.001). For non-contact lens wearers, CoNS was the most frequently reported pathogen (37%), followed by *S.aureus* (18%). The second most prevalent aetiology was severely ill or blind (SI/B) eye with 53 species, equivalent to 24% of the total findings and ocular surface disorder (OSD) was the third most common aetiology with 29 species, corresponding to 13% of the total findings.

**Table 1:** Distribution of laboratory reported organisms and clinical aetiology in the 190 patients with bacterial keratitis

	CLW	ELD	HSV	ID	OSD	PKP	SI/B	OT/FB	BL	Unknown	Total
**GRAM +**											
CoNS^1^	27		5	2	10	7	17	9	3	8	88
*S. aureus*	7	3	2	1	4	2	11		1	7	38
*C. acnes*	5	1	3	1	3	1	3	3	2		22
Alpha-hemolytic streptococci^2^	3				5	3	4	2	2	1	20
*S. pneumoniae*	2			1	3		1	2		2	11
*Corynebacterium* spp.^3^					1		1	1			3
Beta-hemolytic streptococci^4^						1	1				2
Miscellaneous Gram-positive^5^						1	3				4
**Total (** *n* **)**	**44**	**4**	**10**	**5**	**26**	**15**	**41**	**17**	**8**	**18**	**188**
**GRAM -**											
*Pseudomonas* spp.^6^	10				1		3			1	15
*Moraxella* spp.^7^	1	2					3			2	8
*Haemophilus* spp.^8^	2						3		1		6
*F. periodonticum*					1						1
Miscellaneous gram negative^9^	1	1			1		3				6
**Total (** *n* **)**	**14**	**3**	**0**	**0**	**3**	**0**	**12**	**0**	**1**	**3**	**36**
**Grand Total**	**58**	**7**	**10**	**5**	**29**	**15**	**53**	**17**	**9**	**21**	**224**

CLW = contact lens wear, ELD = Eyelid disorder, HSV = Herpes Simplex keratitis, ID = Severely immune deficient patient, OSD = ocular surface disorder, PKP = Penetrating keratoplasty, SI/B = Severely ill or blind eye, OT/FB = Ocular trauma or foreign body in the cornea, BL = blepharitis

Bacteria are reported as species or sub-species or grouped after Gram stain properties and reported as described below.

*1: Staphylococcus capitis*,* Staphylococcus caprae*,* Staphylococcus epidermidis*,* Staphylococcus haemolyticus*,* Staphylococcus hominis*,* Staphylococcus lugdunensis*,* Staphylococcus saccharolyticus*,* Staphylococcus saprophyticus*,* Staphylococcus warneri*,* coagulase-negative staphylococci* not specified.

*2: Alphastreptococcus mitis*,* Alphastreptococcus salivarius*,* Streptococcus anginosus*,* Streptococcus oralis. Streptococci* not specified.

*3: Corynebacterium amycolatum*,* Corynebacterium pseudodiphtheriticum*,* Corynebacterium* not specified.

*4: Betahemolytic streptococci* group G, *Streptococcus agalactiae* (GBS).

*5: Bacillus cereus*,* Enterococcus faecalis.*

*6: Pseudomonas aeruginosa*,* pseudomonas* not specified.

*7: Moraxella catarrhalis*,* Moraxella nonliquefaciens*,* Moraxella* not specified.

*8: Haemophilus influenzae*,* Haemophilus paraprophilus.*

*9: Acinetobacter*,* Enterobacter agglomerans*,* Kingella kingae*,* Proteus mirabilis.*

**Table 2:** A summary of bacterial species causing bacterial keratitis and antibiotic susceptibility in key regions

	CoNS	S. Aureus	Pseudomonas spp.
Sweden, Current study / Sagerfors, Ejdervik-Lindblad [9]			
**Frequency**,** %**	39/40	17/15	7/7
**Susceptibility**,** %**			
Fluoroquinolones	88/-	97/88	100/-
Chloramphenicol	95/-	100/-	0/-
Beta-lactams	83–84/-	100/98	94–100/-
Aminoglycosides	89/-	100/-	100/95
North America, Tam, Côté [24] / Jin, Parker [25]			
**Frequency**,** %**	37/16	15/3	10/21
**Susceptibility**,** %**			
Fluoroquinolones	-/73	-/100	91/100
Chloramphenicol	53/87	96/100	100/100
Beta-lactams	-/-	-/-	-/-
Aminoglycosides	-/100	100/100	97/100
United Kingdom, Ting, Ho [26] / Moledina, Roberts [27]			
**Frequency**,** %**	13/9	16/13	24/30
**Susceptibility**,** %**			
Fluoroquinolones	90/67	91/95	99–100/94
Chloramphenicol	-/73	-/82	-/36
Beta-lactams	-/0	100/0	-/96
Aminoglycosides	93/94–100	100/100	99–100/97–98
Germany and Italy, Roth, Goerke [28] / Grandi, Bianco [29]			
**Frequency**,** %**:	14/18	16/20	18/26
**Susceptibility**,** %**			
Fluoroquinolones	50–89/44–79*	95–100/59–84*	60–99/73–94*
Chloramphenicol	-/68*	-/ 78*	-/-
Beta-lactams	77/-	-/-	-/-
Aminoglycosides	79/38–78*	96/54–86*	96–100/71–85*
Asia, Ahmed, Mishra [30] / Xu, Guo [31]			
**Frequency**,** %**	61/58	14/21	14/-
**Susceptibility**,** %**			
Fluoroquinolones	-/45–47	30–60^†^/81	45–60/-
Chloramphenicol	-/-	70^†^/-	-/-
Beta-lactams	-/-	-/-	40–80/-
Aminoglycosides	-/89	70^†^/81	40–70/-

CoNS: coagulase negative staphylococci

*: Overall susceptibility results for all ocular infections included in the study; Conjunctivitis, Dacryocystitis, Keratitis, Endophthalmitis

†: Susceptibility data reported for all types of Staphylococci found in the study, not S.A exclusively

- no data reported

We defined a sub-group of 42 (22%) subjects with glaucoma. In glaucoma cases, BK was associated with SI/B in 23 subjects (54% of the glaucoma sub-group) or OSD in 11 subjects (26%). The most common species in the glaucoma sub-group were CoNS (15 subjects, 36%) and *S. aureus* (11 subjects, 26%). The results showed that SI/B as well as OSD were significantly more common in subjects with glaucoma when compared to the remaining sample (SI/B: *chi-square test*, p = < 0.001; OSD: *chi-square test*, *p* = 0.003). The OR for SI/B was 9.33 with a 95% CI of 4.23–20.60 and the OR for OSD was 3.69 with a 95% CI of 1.51-9.00, both are statistically significant. There was no difference between glaucoma and non-glaucoma subjects regarding bacterial species found in the samples (CoNS, *p* = 0.342; *S. aureus*, *p* = 0.213).

### Results of antibiotic susceptibility

Table 3 summarizes the susceptibility results for antibiotic categories, excluding beta-lactams that are reported in Table 4. Susceptibility for Gram-positives varied between 88 and 100% and for Gram-negatives between 70 and 100%. Alpha-hemolytic streptococci and *S. pneumoniae* are naturally resistant to aminoglycosides and were hence reported resistant for tobramycin in all cases.

Fluoroquinolones are the first line of recommended treatment and most of the tested isolates were susceptible to ciprofloxacin (156 out of 173). One isolate was resistant to ciprofloxacin but was susceptible to levofloxacin. Resistance to fluoroquinolone was reported for 10 out of 82 tested CoNS (9 isolates for ciprofloxacin and 1 isolate for both ciprofloxacin and levofloxacin) and *S. pneumoniae* was reported “I” for ciprofloxacin in 4 out of 8 tested isolates according to the breakpoint tables valid at the time. Only one *S. aureus* isolate showed reduced susceptibility to ciprofloxacin (fluoroquinolones). Chloramphenicol is a recommended treatment in combination with fluoroquinolones, and the susceptibility was high, with 145 out of 155 tested isolates being susceptible. Resistance to chloramphenicol was detected in four CoNS isolates and aminoglycoside resistance was reported in 9 of 84 isolates for the CoNS. *Pseudomonas* spp. was resistant to chloramphenicol in all 5 cases, which is in line with the expected resistance profile for the species and is typically stated in treatment guidelines. For *Pseudomonas* spp. the current treatment guidelines recommend a combination of fluoroquinolones and aminoglycosides. All *Pseudomonas* spp. in this study tested susceptible to both antibiotics. Vancomycin is the last line of treatment and were the least frequently tested antibiotic, all 6 tested isolates were CoNS, and they were all susceptible.

Beta-lactam antibiotics are usually second line of treatment and susceptibility for these antibiotics varied between 89 and 92% for Gram-positives and 67 to 100% for Gram-negatives respectively, a complete summary is given in Table 4. There was resistance for 2nd generation cephalosporins in one *H. influenzae* isolate out of two reported by the laboratory. In Gram-positive isolates, beta-lactam resistance was observed for CoNS, all other Gram-positive species were reported susceptible for all beta-lactam antibiotics. Notably, all 38 *S. aureus* isolates in the study were susceptible to cephalosporins of all tested generations, thereby excluding the MRSA phenotype.

Table 2 summarizes a selection of recent publications that report their result in a format comparable to the current study. The table includes a selection of pathogens together with antibiotic susceptibility results from antibiotics classes that are the first- and second-line treatment in Sweden. For more comprehensive reviews about antibiotic resistance, we refer readers to other publications in our reference list [9, 24–31].

**Table 3:** Cultured organisms and antibiotic susceptibility for all categories of antibiotics tested except for beta-lactams

	Cultured organisms		Aminoglycoside			Chloramphenicol			Fluoroquinolone				Vancomycin		
	*n*	%▪	S	R	%*	S	R	%*	S	I	R	%*	S	R	%*
**GRAM +**															
CoNS	88	39	75	9	89	76	4	95	72	-	10	88	-	-	-
*S. aureus*	38	17	40	0	100	37	0	100	37	-	1	97	-	-	-
*C. acnes*	22	10	-	-	-	1	0	100	-	-	-	-	-	-	-
Alpha-hemolytic streptococci^1^	20	9	0	3	0	3	0	100	3	-	0	100	-	-	-
*S. pneumoniae*	11	5	0	4	0	11	0	100	4	4	0	50	1	0	100
Other Gram-positive bacteria^2^	9	4	2	0	100	3	0	100	7	0	0	100	5	0	100
**GRAM + total**	**188**	**84**	**117**	**16**	**88**	**134**	**4**	**97**	**123**	**4**	**11**	**89**	**6**	**0**	**100**
**GRAM -**															
*Pseudomonas* spp.	15	7	16	0	100	0	5	0	16	-	0	100	-	-	-
*Haemophilus* spp.	6	2	4	0	100	5	0	100	5	-	1	83	-	-	-
Other Gram-negative bacteria^3^	15	7	9	0	100	9	1	90	13	0	0	100	-	-	-
**GRAM – total**	**36**	**16**	**29**	**0**	**100**	**14**	**6**	**70**	**34**	**0**	**1**	**97**	**-**	**-**	
**Grand total**	**224**	**100**	**146**	**16**	**90**	**145**	**10**	**94**	**157**	**0**	**16**	**91**	**6**	**0**	**100**

^1^: *Alphastreptococcus mitis*, *Alphastreptococcus salivarius*,* Streptococcus anginosus*, *Streptococcus oralis.* streptococci not specified

^2^: *Bacillus cereus*, Beta-hemolytic streptococci, *Corynebacterium* spp., *Enterococcus faecalis*

^3^: *Acinetobacter* spp., *Enterobacter agglomerans*,* Fusobacterium periodonticum*, *Kingella kingae*, *Moraxella* spp., *Proteus mirabilis*

S: susceptible, I: intermediate, R: resistant

▪: Percentage of all organisms

*: Percentage of organisms susceptible for specific antibiotic, calculated by formula: (*n* susceptible cases/n total tested cases) x100

-: no laboratory result reported

**Table 4:** Cultured organisms and antibiotic susceptibility for beta-lactams

	Cultured organisms		Cephalosporin 1st			Cephalosporin 2nd			Cephalosporin 3rd			Carbapenem			
	*n*	%▪	S	R	%*	S	R	%*	S	R	%*	S	I	R	%*
**GRAM +**															
CoNS	88	39	69	14	83	68	14	83	69	14	83	64	1	11	84
*S. aureus*	38	17	40	0	100	38	0	100	40	0	100	30	-	0	100
*C. acnes*	22	10	-	-	-	-	-	-	-	-	-	22	-	0	100
Alpha-hemolytic streptococci^1^	20	9	-	-	-	1	0	100	13	0	100	14	-	0	100
*S. pneumoniae*	11	5	-	-	-	1	0	100	2	0	100	3	-	0	100
Other Gram-positive bacteria^2^	20	9	-	-	-	-	-	-	1	0	100	5	0	0	100
**GRAM + total**	**188**	**84**	**109**	**14**	**89**	**108**	**14**	**89**	**125**	**14**	**90**	**138**	**1**	**11**	**92**
**GRAM -**															
*Pseudomonas* spp.	15	7	-	-	-	-	-	-	16	0	100	15	1	0	94
*Haemophilus* spp.	6	2	-	-	-	1	1	50	1	0	100	-	-	-	-
Other Gram-negative bacteria^3^	21	9	-	-	-	1	0	100	7	0	100	8	0	0	100
**GRAM – total**	**36**	**16**	**-**	**-**	**-**	**2**	**1**	**67**	**24**	**0**	**100**	**24**	**1**	**1**	**96**
**Grand total**	**224**	**100**	**109**	**14**	**89**	**110**	**15**	**88**	**149**	**14**	**91**	**162**	**2**	**12**	**93**

^1^: *Alphastreptococcus mitis*, *Alphastreptococcus salivarius*,* Streptococcus anginosus*, *Streptococcus oralis.* streptococci not specified

^2^: *Bacillus cereus*, Beta-hemolytic streptococci, *Corynebacterium* spp., *Enterococcus faecalis*

^3^: *Acinetobacter* spp., *Enterobacter agglomerans*,* Fusobacterium periodonticum*, *Kingella kingae*, *Moraxella* spp., *Proteus mirabilis*

S: susceptible, I: intermediate, R: resistant

▪: Percentage of all organisms

*: Percentage of organisms susceptible for specific antibiotic, calculated by formula: (*n* susceptible cases/n total tested cases) x100

-: no laboratory result reported

## Discussion

In the current study we report new surveillance data on bacterial species causing BK, predisposing factors, together with the first comprehensive analysis of antibiotic susceptibility among bacterial species causing infectious BK in Sweden.

The most common Gram-positive group was CoNS and single species was *S. aureus*. The most common Gram-negative bacteria was *Pseudomonas* spp. Predisposing factors for BK were contact lens wear and severely ill or blind eye associated with glaucoma, eye surgery or inflammatory systemic diseases. The isolated bacterial species and predisposing factors of infection found in the current study were in line with results from previous reports from Sweden [9, 32], and from other parts of the world [1, 30, 33]. However, there are reports from, for example, Southeast Asia where most cases of BK among contact lens wearers are caused by *P. aeruginosa* [34–36]. In our study *P. aeruginosa* accounted for 7% of the cases. This is an example of how climate and hygiene routines affect the pathogens underlying corneal infections.

Contact lens wear seems to increase the risk of BK, this finding is in line with other studies, but the exact pathogenesis remains unclear [1, 9, 40, 41]. The most common bacteria associated with contact lens wear, for example *Staphylococcus*, *Cutibacterium* and *Pseudomonas*, have also been identified at genus level in normal ocular surface microbiota [42, 43]. Therefore, contact lens-related keratitis seems unrelated to exposure to atypical bacterial species associated with contact lenses. We speculate that contact lenses cause superficial epithelial damage which facilitates commensal bacteria infecting the cornea. Epithelial damage can be caused by, for instance, corneal hypoxia due to contact lens wear, foreign bodies such as foreign particles or eyelashes under the contact lens [44] or insertion and removal of the contact lens [42, 45–47]. Non-compliance with contact lens wear such as extended hours of use or sleeping with contact lenses is common, and this impair the natural renewal of the corneal epithelium leading to extra susceptibility to bacterial invasion [48]. Poor hygiene with contact lenses and their storage case can increase bacterial growth and biofilm formation, leading to excessive bacterial concentration in the cornea [49, 50].

Subjects with severely ill or blind eye and/or ocular surface disease are at greater risk of infection, due to compromised ocular surface integrity [12]. In the glaucoma sub-group, it was 9.3 times more likely to have SI/B and 3.7 times more likely to have OSD compared with those without glaucoma. Assuming that glaucoma precedes the keratitis and the OSD, the OSD can be caused by toxicity of ingredients within the glaucoma drops that functions as preservatives or increase the permeability of the cornea [11]. Glaucoma drops can also be contaminated by bacteria, particularly after repeated self-applications, and these combined effects increase the risk of infection [12, 13]. Having a diagnosis of glaucoma seems a risk factor for bacterial infection and perhaps patients should receive special advice about their increased susceptibility to corneal infections.

Pathogens detected in the current study were susceptible to the spectrum of antibiotics tested, and the current clinical recommendations for antibiotic prescription in Sweden seem well targeted for most cases of bacterial keratitis investigated. These findings are in contrast with studies from other countries showing increasing antibiotic resistance, especially among Gram- negative pathogens [4, 29, 30]. In contrast with other parts of word such as UK, Italy and India [27, 29, 30] where bacteria show significant resistance to chloramphenicol, our study revealed high antimicrobial susceptibility to chloramphenicol. A possible explanation for preserved susceptibility is that, in Sweden, all antibiotics are “prescription-only” and there is great awareness among the population and health professionals regarding antibiotic resistance. As a result, Sweden has substantially reduced the use of antibiotics in animals and humans [51]. Our findings are consistent with the low reported antibiotic resistance available from surveillance data for both Sweden and other Nordic countries [52, 53].

According to Swedish treatment guidelines, suspected *P. aeruginosa* caused BK should be treated with a combination of aminoglycosides and fluoroquinolones [15] and chloramphenicol should be avoided [54]. Results from the current study are in agreement with past studies from Sweden or Europe [4, 9] and indicate that all *Pseudomonas* spp. were susceptible to the recommended antibiotics, and resistant to chloramphenicol. This result confirms the validity of the current guidelines.

The second line of treatment for BK in Sweden is the use of beta-lactams, namely cephalosporins or carbapenems. Findings given in Table 3 revealed a high susceptibility to beta-lactams among the bacterial species found in our study which is in line with previous reports [55]. These results confirm that the current second-line treatment recommendations for antibiotic prescription remain valid.

*S. aureus* was detected in 38 cultures, but none of them were reported methicillin resistant [11]. Surveillance data from blood cultures performed during the same period as the current study showed a proportion of MRSA in Sweden of 1.1-2.0% [53, 56]. The Nordic MRSA surveillance study reported an increase in MRSA occurrence; however, these results include findings from both screening (carriage) and infections [57], possibly overestimating expected MRSA rates in BK. In some parts of the USA and Mexico, MRSA in ocular samples can be as high as 40% [16, 58]. In contrast, the rates of MRSA in Sweden, Canada (1.3%) [59] and UK (3%) are low [60]. Another reason for low MRSA is that it is designated as a compulsory notifiable disease in Sweden, and infections are retraced to prevent spreading [61]. The findings of our study are encouraging and show that the measures in place in Sweden are preventing MRSA.

The current study has both strengths and limitations. A strength is that it provides the only detailed information about antibiotic susceptibility for bacterial species detected in BK allowing guidelines and trends in our region to be assessed. Limitations of the current study include the lack of patient follow-up, thus precluding an analysis of clinical outcomes of treatment including those with culture negative lab results; and the retrospective design of the study resulting in an inability to account for missing data in some patient records. Larger studies of keratitis involving more regions of Sweden are necessary to determine if regional differences exist, including occurrence of other forms of keratitis such as those caused by acanthamoeba or fungi, preferably using a metagenomic approach with next-generation sequencing for more efficient and detailed pathogen detection [62]. The connection between infectious keratitis and glaucoma is also a topic that should be further investigated.

## Conclusion

In real world practice, most cases of suspected or clinically diagnosed bacterial keratitis are treated without sending corneal or conjunctival samples to the microbiology laboratory for confirmation. Our results revealed a positive rate of BK of 59% for the samples sent to the laboratory, which indicate that the criteria for laboratory testing is sensitive. A subpopulation of 22% of patients had glaucoma concomitant with BK, representing an association that requires further investigation. CoNS were the most common bacterial species, followed by *S. aureus*. There was a high susceptibility of the bacterial species to the recommended antibiotics, and we failed to find cases of MRSA. These findings indicate that it is likely that patients, regardless of culture-confirmed infection, received and are receiving the correct treatment for bacterial keratitis.

## Data Availability

No datasets were generated or analysed during the current study.

## Appendix A

### Sample collection

During direct inoculation, corneal samples were undertaken after the installation of topical unpreserved anaesthesia from the infiltrate with a sterile cotton swab and a 21-gauge sterile needle. The swab was then used to directly inoculate half of the area of two solid media (hematin and blood agar) and a 21-gauge sterile needle was used to inoculate the other half of the two solid media. For indirect inoculation, the same 21-gauge needle was submerged in fastidious anaerobic broth in a sterile fashion, which was later subcultured onto agar plates for both anaerobic and aerobic incubation at the laboratory.

Conjunctival samples were taken with the indirect inoculation method. Care was taken to avoid touching eyelids and a sterile cotton swab was used after the instillation of preservative-free topical anaesthetic drops. The cotton swab was brushed against the conjunctival surface. The tip of the cotton swab was submerged in transport media and sent to the laboratory.

## Appendix B

### Culture records

The methods used by the laboratory have been assured by accreditation from the Swedish national accreditation body (SWEDAC). The methodology has changed over time according to relevant national guidelines and current reference methods.

For data reporting, bacteria were either reported as:

- individual species and sub-species (*Cutibacterium acnes*, *Fusobacterium peridonticum*, *Staphylococcus aureus*, *Streptococcus pneumoniae*) or.
- at species level (*Corynebacterium* spp., *Pseudomonas* spp., *Moraxella* spp. and *Haemophilus* spp.) or,
- grouped as following: - coagulase-negative staphylococci, CoNS (*S. capitis*,* S. caprae*,* S. epidermidis*,* S. haemolyticus*,* S. hominis*,* S. lugdunensis*,* S. saccharolyticus*,* S. saprophyticus*,* S. warneri* and non-specified CoNS), - a-hemolytic streptococci (*S. mitis*,* S. salivarius*,* S. anginosus*,* S. oralis* and non-specified a-hemolytic streptococci), - b-hemolytic streptococci (*S. agalactiae* and group G b-hemolytic streptococci).

Other infrequent findings were divided into miscellaneous Gram-positives (*Bacillus cereus*,* Enterococcus faecalis*) and miscellaneous Gram-negatives (*Acinetobacter*,* Enterobacter agglomerans*, *Kingella kingae*, *Proteus mirabilis*).

## Abbreviations

BK: Bacterial keratitis
BL: Blepharitis
CLW: Contact lens wear
CoNS: Coagulase-negative staphylococci
ELD: Eyelid disorder
HSV: Herpes Simplex keratitis
ID: Severely immune deficient patient
IK: Infectious keratitis
MRSA: Methicillin-resistant *Staphylococcus aureus*
OSD: Ocular surface disorder
OT/FB: Ocular trauma or foreign body in the cornea
PKP: Penetrating keratoplasty
SI/B: Severely ill or blind eye
